# Past and Contemporaneous Otolith Fingerprints Reveal Potential Anthropogenic Interferences and Allows Refinement of the Population Structure of *Isopisthus parvipinnis* in the South Brazil Bight

**DOI:** 10.3390/biology11071005

**Published:** 2022-07-03

**Authors:** Natasha Travenisk Hoff, June Ferraz Dias, Edgar Pinto, Agostinho Almeida, Rafael Schroeder, Alberto Teodorico Correia

**Affiliations:** 1 Laboratório de Ecologia da Reprodução e do Recrutamento de Organismos Marinhos, Departamento de Oceanografia Biológica, Instituto Oceanográfico, Universidade de São Paulo (USP), Praça do Oceanográfico, 191, São Paulo 05508-120, Brazil; natasha.hoff@usp.br (N.T.H.); junedias@usp.br (J.F.D.); 2 Programa de Pós-Graduação em Oceanografia, Universidade de São Paulo (USP), Praça do Oceanográfico, 191, São Paulo 05508-120, Brazil; 3 Centro Interdisciplinar de Investigação Marinha e Ambiental (CIIMAR/CIMAR), Terminal de Cruzeiros do Porto de Leixões, Avenida General Norton de Matos S/N, 4450-208 Matosinhos, Portugal; schroederichthys@gmail.com; 4 LAQV/REQUIMTE, Faculdade de Farmácia da Universidade do Porto, 4050-313 Porto, Portugal; ecp@ess.ipp.pt (E.P.); aalmeida@ff.up.pt (A.A.); 5 Departamento de Saúde Ambiental, Escola Superior de Saúde, P. Porto. Rua Dr. António Bernardino de Almeida 400, 4200-072 Porto, Portugal; 6 Laboratório de Estudos Marinhos Aplicados, Escola do Mar, Ciência e Tecnologia, Universidade do Vale do Itajaí (UNIVALI), Rua Uruguai 458, Itajaí 88302-901, Brazil; 7 Faculdade de Ciências da Saúde da Universidade Fernando Pessoa (FCS/UFP), Rua Carlos Maia 296, 4200-150 Porto, Portugal; 8 Instituto de Ciências Biomédicas Abel Salazar da Universidade do Porto (ICBAS-UP), Rua de Jorge Viterbo Ferreira 228, 4050-313 Porto, Portugal

**Keywords:** Sciaenidae, otolith fingerprints, population units, temporal shifts

## Abstract

**Simple Summary:**

Otolith geochemical signatures were important tools used to investigate the population of commercially exploited fish species. Historical and contemporary otolith samples of *Isopisthus parvipinnis*, Bigtooth corvina, an economically and ecologically important Brazilian fish species, collected in five subareas [São Paulo: North—NSP, Center—CSP and South—SSP; Paraná (PR) and Santa Catarina (SC)] of the shallow waters off the coast of the South Brazil Bight were used in this study. Univariate and multivariate statistical analyses showed spatial differences in otolith chemical composition over the years, suggesting that long-term temporal variability in oceanographic conditions, anthropogenic influence, and climate change on this coastal ecosystem influenced the geochemical signatures. Moreover, these results also confirm that *I. parvipinnis* is not a single and homogeneous fish stock in this geographic area, supporting the existence of a metapopulation structure scenario and corroborating previous studies that used alternative, complementary phenotypic tags.

**Abstract:**

In this study, otolith geochemical signatures (Element:Ca ratios) were used to investigate the long-term spatial shifts of the population structure of *Isopisthus parvipinnis*, Bigtooth corvina, an economically and ecologically important Brazilian fish species. Two-hundred and ninety-seven juvenile individuals from historical (1975) and contemporary (2018/2019) samples were collected in five subareas [São Paulo: North—NSP, Center—CSP and South—SSP; Paraná (PR) and Santa Catarina (SC)] of the shallow waters off the coast of the South Brazil Bight were analyzed. The main informative single elements were Co:Ca, Cu:Ca, Li:Ca, Mg:Ca, Mn:Ca, Ni:Ca, Na:Ca, and Rb:Ca. Multivariate analysis showed spatial differences in otolith chemical composition over the years. Samples from 1975 presented an overall low reclassification rate (58%), suggesting the existence of two population units: (1) SP + PR; and (2) SC. However, samples from 2018/2019 discriminated four distinct population units with a good overall reclassification (80%): (1) NSP; (2) CSP; (3) SSP + PR; and (4) SC. This spatial differentiation on the geochemical signatures probably reflects the effects of long-term temporal variability in oceanographic conditions, anthropogenic influence, and climate change on this coastal ecosystem. The data also corroborate and refines the population structure scenario of *I. parvipinnis* recently described using complementary phenotypic tags.

## 1. Introduction

A proper understanding of the fish population structure and dynamics is essential for the rational management of a fishery [1]. Since population units may respond differently to exploitation, they must be managed separately to optimize their maximum sustainable yield [2]. Accurate knowledge of the population structure can have significant impacts on the rational and sustained management of fish stock, including the necessary adjustments in response to fishing pressure and environmental changes, which play a key role in the species’ persistence [3]. Several natural tags, such as otolith’s shape and its chemical composition, body meristic and morphometric characters, and the presence and prevalence of parasites, among others, have been commonly used in fisheries biology, providing evidence for stock discreteness [4,5,6]. Otolith microchemistry, for instance, can be pointed to as a successful approach not only to infer about fish population structure but also to help us to solve questions such as natal origin, migration patterns, habitat use, and connectivity, namely, where environmental heterogeneity exists [7,8,9]. Indeed, coastal systems embrace an inherent variability of abiotic factors that are under the influence of human action and climate change [10,11,12]. The Brazilian coast, in particular, encompasses a great variety of estuarine and oceanic ecosystems that can provide different chemical signatures for fish otoliths. Therefore, otoliths were successfully used to assess the estuarine dependency and habitat use of *Micropogonias furnieri* Whitemouth croaker (Sciaenidae) [13], *Centropomus parallelus* Fat snook (Centropomidae) [14], and *Cathorops spixii* Madamango sea catfish (Arriidae) [15] in the South Brazil Bight (SBB: 23° S to 29° S of latitude); connectivity and population structure of *Stegastes fuscus* Brazilian damsel (Pomacentridae) [7] and *Abudefduf saxatilis* Sergeant-major (Pomacentridae) [8] from coastal systems of São Paulo, Paraná and Santa Catarina (25° S to 26.5° S); stock structure and life-time movements of *Chaetodipterus faber* Atlantic spadefish (Ephippidae) from Espírito Santo to Santa Catarina states (20° S to 28° S) [9,16]; and fish stocks and nursery areas of *Genidens genidens* Guri sea catfish (Ariidae) along the southeastern and southern coast of Brazil (21.6° S to 25.5° S) [17].

Drums and croakers are fishes belonging to the Sciaenidae family (order: Acanthuriformes) with over 250 species worldwide [18], from which more than 20 can be found in the estuarine and coastal waters of the western Atlantic Ocean [19,20]. They are also one of the most important demersal fisheries resources in the shallow waters of the South Brazil Bight (SSB) [21,22,23]. The Bigtooth corvina, *Isopisthus parvipinnis*, is widely distributed along the western Atlantic Ocean, from Costa Rica to Brazil, being caught as bycatch of the small-scale artisanal fisheries, mainly in the Brazilian Southeast-South region [24]. It represents 6% of the artisanal discards in south and southeastern Brazil [25], and depending on its size, it can be used as bait or sold in a mixture of different fish species or individually [26]. *I. parvipinnis* is a seasonal species usually found on the inner continental shelf at 23° S [27]. *I. parvipinnis* is mainly a piscivorous fish, and its feeding regime varies with season and ontogeny [28,29]. During spring and summer, individuals sporadically move to the most open areas of the Guaratuba Bay, southern Brazil, and feed on fish and crustaceans. During this period, the spawning occurs either in the mangrove or in the open sea next to the state of Paraná. However, in autumn and winter, an important part of the population of *I. parvipinnis* enters into channels and pools, and its diet changes to become entirely based on fish [30]. The reproductive period of the species in the northern Santa Catarina (southern Brazil) occurs mainly in spring and summer, which coincides with the season closure of the shrimp fishery [31]. Fish population recruitment appears to occur between summer and autumn to winter in northeast Brazil [32]. The population structure of *I. parvipinnis* was recently studied in southeast–south Brazil through the study of the otolith shape [33] and body geometric morphometrics [34], revealing a complex metapopulational scenario with discrete population units.

The aim of this work was twofold. Firstly, to investigate the long-term stability of the population structure of *I. parvipinnis* in the SBB using trace elements recorded in otoliths collected in a temporal window of 43 years apart (1975 vs. 2018/2019); and, secondly, to evaluate for the first time, the spatial-temporal variations in the chemical composition of otoliths, as environmental time-lag recorders, taking into consideration the long-term effects of human activities and climate change on the marine ecosystems.

## 2. Materials and Methods

### 2.1. Fish Sampling and Otolith Preparation

**Figure 1:**
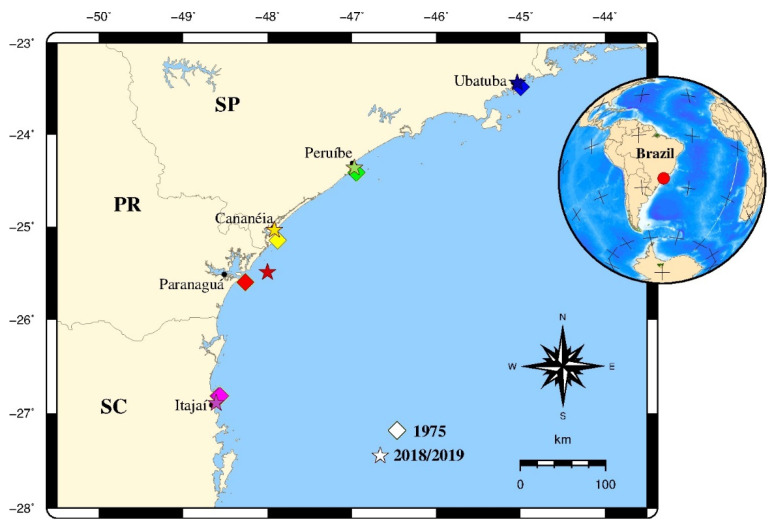
Sampling locations of *Isopisthus parvipinnis* juvenile individuals collected in 1975 (squares) and 2018/2019 (stars) in the South Brazil Bight from 23° S to 26.9° S, between São Paulo and Santa Catarina states. Legend: North of São Paulo (NSP), Center of São Paulo (CSP), South of São Paulo (SSP), Paraná (PR), Santa Catarina (SC).

Two-hundred and ninety-seven individuals [all juveniles, since their total length (TL: 66–139 mm) was less than the length at first maturity (159 mm) according to [32]] were caught from commercial and research vessels in the shallow waters of the SBB (from 23° S to 29° S) in five sampling areas: Northern (NSP), Center (CSP), and Southern (SSP) of São Paulo, Paraná (PR) and Santa Catarina (SC) (Figure 1 and Table 1). The individuals were collected (i) between September and November 1975 (*n* = 149) by the Oceanographic Institute of the University of São Paulo during the Nectonic Fauna research project (FAUNEC) and thereafter archived at the ColBIO (Coleção Biológica Edmundo Ferraz Nonato IO-USP) (for more details see [35]); and (ii) between September 2018 and May 2019 (*n* = 148) by local fishermen, preserved on ice and thereafter processed at the laboratory. For both sampling periods, an otter trawl was used.

The individuals selected for otolith elemental analysis were restricted, as much as possible, to a narrow size range for each year, with the exception of SC to 2018/2019 (Table 1), to minimize phenotypic variations resulting from ontogenetic processes [36]. The fish were identified through meristic and morphometrics characters to the species level [37]. The total length (TL, 0.1 mm) and body weight (W, 0.01 g) were measured for each individual. Sagittal otoliths were carefully extracted, cleaned from organic tissues, washed with distilled water, dried and stored in plastic tubes.

The right otoliths were cleaned in an ultrasonic bath using ultrapure water (H_2_O Milli-Q-Water: > 18.2 MΩ.cm at 25 °C) for five minutes, followed by immersion in 3% (*v/v*) ultrapure hydrogen peroxide (H_2_O_2_: Honeywell Fluka, TraceSELECT™, ≥30.0%) for 15 min to remove biological residues, and thereafter superficially decontaminated in 1% (*v/v*) nitric acid (HNO_3_: Honeywell Fluka, TraceSELECT™, ≥69.0%) for 10 s [38]. Thereafter, the otoliths were rinsed by triple immersion in ultrapure water (H_2_O: Milli-Q-Water > 18.2 MΩ.cm at 25 °C) for five minutes, dried in a laminar flow cabinet hood, and stored in decontaminated plastic tubes [39].

### 2.2. Otolith Elemental Analysis

Otoliths were weighed on an analytical balance (OM, 0.0001 g), dissolved for 15 min in 0.1 mL of ultrapure nitric acid (HNO_3_: Honeywell Fluka, TraceSELECT™, ≥69.0%), and diluted with ultrapure water (H_2_O: Milli-Q-Water > 18.2 MΩ.cm at 25 °C) to a final volume of 5.0 mL (2% of HNO_3_ *v/v* and 0.2% of TDS *m/v*) [9].

Multi-elemental analyses of trace elements (µg/L in liquid sample) were performed by Solution-Based Inductively Coupled Plasma Mass Spectrometry (SB-ICP-MS) using an iCAPTM Q instrument (Thermo Fisher Scientific, Bremen, Germany) equipped with a concentric glass nebulizer, a Peltier-cooled baffled cyclonic spray chamber, a standard quartz torch and a two-cone interface design (sample and skimmer nickel cones). High-purity (99.9997%) argon (Gasin II, Leça da Palmeira, Portugal) was used as the nebulizer and plasma gas. The equipment control and data acquisition were performed on Qtegra software (Thermo Fisher Scientific). Indium(115In), Scandium (45Sc), Terbium (159Tb), and Yttrium (89Y) were monitored as internal standards to minimize the effect of plasma fluctuations or different nebulizer aspiration rates among the samples [6]. The limits of detection (LOD) were calculated as the concentration corresponding to three times the standard deviation of 10 sample blanks. However, minor elements such as calcium, sodium, and strontium, because of their high concentrations in the aragonite matrix (mg/L in the liquid sample), which may precipitate in the nebulizer or overload the plasma, were determined by a Flame Atomic Absorption Spectrometry (FAAS) instrument (Perkin Elmer, Überlingen, Germany). Moreover, with low-mass otoliths, such as the otoliths of *I. parvipinnis*, and when we are already working with a small liquid sample volume (5 mL), further dilution of the samples to evaluate all the elements (minor and trace) in the ICP-MS, would necessarily lead to the loss of some trace informative elements [40]. In any case, analytical control was performed in both techniques. In order to avoid possible sequence effects, all samples (ICP-MS and FAAS) were analyzed randomly.

A preliminary analysis detected 20 trace elements, but the concentration of 8 of them (^75^As, ^111^Cd, ^52^Cr, ^98^Mo, ^121^Sb, ^82^Se, ^205^Tl, and ^66^Zn) was consistently below the LOD, and therefore they are excluded from analyses. Twelve elements were above the LOD: ^137^Ba (0.032 μg.L^−1^), ^43^Ca (2.384 μg.L^−1^), ^59^Co (0.001 μg.L^−1^), ^65^Cu (0.020 μg.L^−1^), ^7^Li (0.002 μg.L^−1^), ^26^Mg (0.268 μg.L^−1^), ^55^Mn (0.010 μg.L^−1^), ^23^Na (0.138 μg.L^−1^), ^60^Ni (0.016 μg.L^−1^), ^208^Pb (0.003 μg.L^−1^), ^85^Rb (0.005 μg.L^−1^), and ^88^Sr (0.006 μg.L^−1^). These elements, commonly used for fish population assessment purposes [9,36,41], were considered useful biogeochemical tags for *I. parvipinnis*. NIES CRM 22 (a fish otolith certified reference material from the National Institute for Environmental Studies, Japan) was used for accuracy control, with recovery values between 83% and 90%. The precision of replicate analyses (relative standard deviation) of individual elements was, in general, below 5%. Concentrations of trace elements, originally in μg element.L^−1^ solution, were transformed to μg element.g^−1^ otolith, and finally to μg element.g^−1^ calcium [42].

### 2.3. Statistical Analysis

Prior to statistical analyses, the data were checked for normality (Shapiro–Wilk’s test), homoscedasticity (Levene’s test), and the presence of outliers (Grubbs’ test). The relationship between elemental concentration and fish size (expressed as otolith mass and used as a covariate) was tested with Analysis of Covariance (ANCOVA). For all the element:Ca ratios that showed a negative (Ba:Ca, Li:Ca, Mg:Ca, Mn:Ca, Na:Ca, Pb:Ca, Sr:Ca) or positive (Co:Ca, Cu:Ca, Ni:Ca) relationship with OM (ANCOVA, *p* < 0.05), the individual data were weight-detrended by the subtraction of the common within-group linear slope [36]. Differences in single elemental fingerprints among locations and years were explored by a Two-Way Analysis of Variance (Two-Way ANOVA), respectively, followed by a Tukey’s post-hoc test if significant (*p* < 0.05). Differences in multi-elemental fingerprints among locations and years were tested using an overall and pairwise Permutational Multivariate Analysis of Variance (PERMANOVA) based on the Euclidean distance measure using 9999 random permutations. A Canonical Analysis of Principal Coordinates (CAP) based on Euclidian distances was performed to visualize regional differences in each year. Variables that most contributed to each axis, based on the Pearson correlation (r > 0.50), were displayed in CAP two-dimensional plots. The reclassification accuracy of the discriminant functions for each location was evaluated through the percentage of correctly re-classified individuals to the origin using a leave-one-out cross-validation [33].

All of the statistical analyses were performed using Past—Version 4.03 and PRIMER 7 + PERMANOVA software, with a statistical level of significance (α) of 0.05.

## 3. Results

Six element:Ca ratios (Ba:Ca, Cu:Ca, Li:Ca, Mg:Ca, Mn:Ca, and Sr:Ca) presented significant differences among locations, between years, and for the interaction between them ANOVA, *p* < 0.05, Table 2); but Pb:Ca only resulted in significant differences among locations and years (Two-Way ANOVA, *p* < 0.05, Table 2). The other four element: ratios (Co:Ca, Na:Ca, Ni:Ca and Rb:Ca) did not present any differences between years (Two-Way ANOVA, *p* > 0.05, Table 2), but differences among locations and locations x years were observed (Two-Way ANOVA, *p* < 0.05, Table 2).

**Figure 2:**
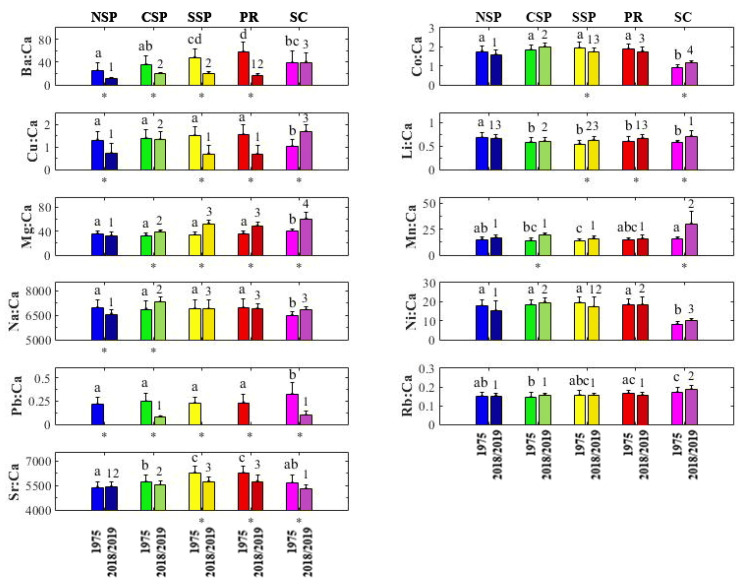
Element:Ca ratios in otoliths of *Isopisthus parvipinnis* collected in the South Brazil Bight in 1975 and 2018/2019. Locations sharing the same letter (year 1975) or number (year 2018/2019) do not show any statistical difference (Two-Way ANOVA, Tukey’s post-hoc tests, *p* > 0.05). Significant time differences in each location are shown by an asterisk (*) (Two-Way ANOVA, Tukey’s post-hoc tests, *p* < 0.05). Data are presented as mean values ± SE. Legend: North of São Paulo (NSP), Center of São Paulo (CSP), South of São Paulo (SSP), Paraná (PR), Santa Catarina (SC).

Li:Ca and Sr:Ca showed significant differences between years in SSP, PR, and SC; but Ba:Ca and Mg:Ca recorded temporal differences for all locations, except SC and NSP, respectively (Tukey’s post-hoc test, *p* > 0.05; Figure 2). 

Regarding the year 1975, six element:Ca ratios distinguished all locations from SC. The former location presented the highest levels of Mg:Ca and Pb:Ca, and the lowest levels of Co:Ca, Cu:Ca, Na:Ca, and Ni:Ca. Moreover, Li:Ca was significantly higher in NSP than in other locations. Ba:Ca and Sr:Ca presented the same patterns, with higher values in SSP and PR. A slight increase in Rb:Ca southwards was also observed. However, Mn:Ca did not show any pattern (Tukey’s post-hoc test, *p* > 0.05; Figure 2). For 2018/2019, Ba:Ca, Mn:Ca, and Rb:Ca presented values significantly higher in SC compared to other locations, while Co:Ca and Ni:Ca presented the lowest values in the same region; Li:Ca was significantly higher in NSP, PR, and SC; Mg:Ca was able to differentiate among locations, with a latitudinal increase in concentration from NSP to SC; Cu:Ca showed values significantly lower for NSP/SSP/PR than for CSP and SC; and Na:Ca presented significant differences between NSP, CSP and SSP/PR/SC; Pb:Ca was only detected in CSP and SC, with much lower values compared to 1975; and Sr:Ca presented the same pattern observed in 1975, with higher values in SSP and PR (Tukey’s post-hoc test, *p* < 0.05; Figure 2).

Regarding the multi-elemental fingerprints, the PERMANOVA analysis showed significant differences between years and among sampling locations and also detected a significant interaction between both factors (overall PERMANOVA, *p* < 0.05; Table 3). Pairwise analysis showed significant differences among all locations for both years, except between SSP and PR, also for both years’ comparisons (pairwise PERMANOVA, *p* > 0.05, Table 3).

**Figure 3:**
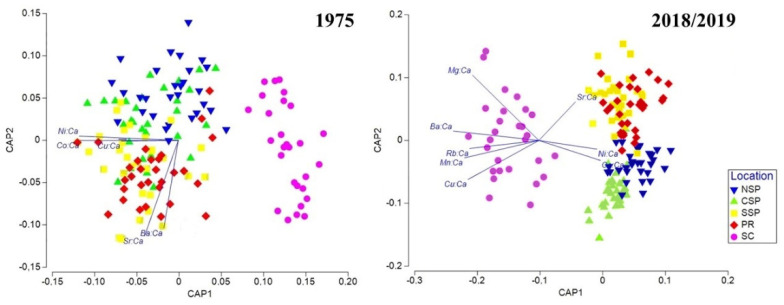
Canonical analysis of principal coordinates (CAP) plots from otoliths chemical composition analysis of *Isopisthus parvipinnis* collected in the five sampling locations in the South Brazil Bight in 1975 and 2018/2019. Legend: North of São Paulo (NSP), Center of São Paulo (CSP), South of São Paulo (SSP), Paraná (PR), and Santa Catarina (SC).

Regarding the historical samples, CAP showed some overlap between samples from SP and PR but a clear distinction between SC samples (Figure 3, 1975). Vector overlays indicated that group separation was primarily driven by Ni:Ca (r = −0.94), Co:Ca (r = −0.93) and Cu:Ca (r = −0.54) on CAP Axis 1, and by Sr:Ca (r = −0.91) and Ba:Ca (r = −0.83) on CAP Axis 2. The leave-one-out cross-validation presented a low overall reclassification success of 58%, highlighting the SC samples that were all fully reallocated to the original location (100%) and the NSP samples that reached 70% of correct reclassification, contrary to CSP and SSP (Table 4).

The overall reclassification success increased to 80% in 2018/2019, with reclassification percentages increased for all sampling locations, mainly in NSP (97%) and CSP (87%, Table 4). Again, samples from SC were all fully reclassified (100%), while SSP and PR samples were reallocated mainly between them. CAP results were similar again, with the three locations from São Paulo being well distinguished but with an overlap between SSP and PR samples (Figure 3, 2018/2019). Vector overlays indicated that group separation was primarily driven by Mg:Ca (r = −0.64), Mn:Ca (r = −0.71), Rb:Ca (Pearson, r = −0.66), Cu:Ca (Pearson, r = −0.68), Ba:Ca (Pearson, r = −0.82), Ni:Ca (r = 0.56), Co:Ca (Pearson, r = 0.59), and Sr:Ca (Pearson, r = 0.35) on CAP Axis 1, and by Mg:Ca (Pearson, r = 0.62), Cu:Ca (Pearson, r = −0.37), Sr:Ca (Pearson, r = 0.37) on CAP Axis 2.3.1.

## 4. Discussion

The otolith elemental composition of *I. parvipinnis* recorded significant regional and temporal variations. All single element: Ca ratios showed significant regional differences within years, but not necessarily for all locations. Regarding the multivariate analysis, the correct reallocation of individuals to the original locations was smaller in 1975 (overall 58%) compared to 2018/2019 (overall 80%), where an increment for the reclassification rates was observed for all locations. Moreover, the SC samples reached a full reclassification success of 100% in both periods. The coastal zone of SC, the southernmost region of the study area, is under the influence of important oceanographic processes: wind-driven South Atlantic Central Water intrusions towards the coast take place in a large portion of the shelf, nearly 28.5° S [43]; and the cold (14–17 °C) and less salty (33.0–34.0) water transported by the Brazil Coastal Current, consisted of waters from the Argentina continental shelf, near the Río de la Plata mouth, and the Brazil-Malvinas Confluence [43,44,45]. As a consequence, SC waters are nutrient-enriched, being classified as mesotrophic, reaching eutrophic conditions during some periods of the year [46,47]. These oceanographic characteristics are very different from the other areas, such as NSP, in which the waters are oligotrophic, which could be reflected in the element:Ca ratios signatures and consequently in the 100% reclassification success for SC.

Taking into consideration the elemental signatures recorded in 1975, two population units were recorded: one including SP and PR states but already suggesting an initial segregation between NSP/CSP and SSP/PR, and SC, a fully isolated population. Similar results were already observed in the past (in the 1970s and 1980s) for other sciaenid species in the SSB inferred from the body and/or otolith shape (e.g., *Cynoscion jamaicensis*, [48,49]; *Macrodon ancylodon*, [50]; *Micropogonias furnieri*, [51]; *Nebris microps*, [52]). However, at present (2018/2019), and 43 years later, four main population units, NSP, CSP, SSP/PR, and SC, were clearly depicted regarding *I. parvipinnis* population structure, as recently suggested by previous studies using alternative approaches [33,34].

The study area, with about 700 km of coastline, covers a wide variety of aquatic ecosystems under the influence of different oceanographic features and anthropogenic activities [53]. NSP and SC are exposed to seasonal intrusions toward the coast by the cold oceanic South Atlantic Central Water (SACW), a water mass transported by the Brazil Current along the Brazilian continental slope [54,55]; CSP is under coastal events, and minor rivers influence; and SSP and PR are under the influence of great estuarine ecosystems that have changed over time as a result of intense human activities [56,57]. These environmental factors can lead to regional geochemical differences or similarities between fish populations [6,9,58]. Moreover, fish physiological dynamics and experiential life-history traits could result in population-specific differences in otolith chemical composition, even in chemically homogeneous conditions [59,60]. Although some general hydrographic processes remain unchanged over time, such as the SACW intrusions, the biotic integrity of SBB’s coastal ecosystems is changing [61,62,63]. Therefore, it would be challenging to pinpoint all the causes behind the regional (i.e., spatial) and temporal otolith chemistry variability recorded in the hereby study. Additionally, the incorporation of minor and trace elements in fish otoliths depends on many factors, including their concentration and bioavailability in water, physiological processes, individual somatic growth, and the affinity of the aragonite matrix otolith for the different elements [60,64].

All element:Ca ratios presented significant regional differences in 2018/2019, limited to Ba:Ca, Co:Ca, Cu:Ca, Ni:Ca, and Sr:Ca in 1975. Barium (Ba) and strontium (Sr) are considered the elements that best represent the surrounding water composition since they may pass through calcium channels, directly replacing them in the calcium carbonate matrix, which results in higher abundances in otoliths [60]. These elements usually present a direct relationship with salinity, negative for Ba:Ca and positive for Sr:Ca, which could explain higher values of Ba:Ca in SSP and PR, but not the Sr:Ca patterns observed for both periods, which seems to be also influenced by ontogenetic processes, such as growth and spawning [65,66,67]. Likewise, sodium (Na) is also essential for cellular processes (e.g., N, K—ATPase), with high physiological response in its regulation, but the influence of environmental factors in the otolith absorption remains somewhat unclear [36,68,69], as well as its profile in this study area. Lithium (Li) incorporation into biogenic calcium carbonates involves little biological control, also replacing the Ca in the aragonite/calcite matrices directly [7,70]; thus, Li:Ca is tied to environmental influences such as salinity, temperature, upwelling conditions, or primary productivity, rather than to otolith specificities [59,71,72,73]. So, a possible explanation for higher Li:Ca values in NSP and SC in 1975 and 2018/2019, respectively, could be related to the new primary production accompanying upwelling events in these locations. The presence of cobalt (Co) and nickel (Ni), as well as zinc (Zn) and lead (Pb), is primarily through their physiological roles as biomolecule co-factors rather than as a result of the environment [74]; regardless, Co:Ca and Ni:Ca, were significantly lower in both periods for SC. Magnesium (Mg) is unrelated to either temperature or salinity in marine fish otoliths [75], but it is negatively correlated with metabolic processes and growth rate [59,72]; therefore, it is expected that changes in metabolic rates caused by environmental fluctuations (e.g., temperature and food resources variations) could cause alterations in elemental assimilation rates [72]. Studies about manganese (Mn) are still contradictory. It is unclear if it reflects changes in the physicochemical environment experienced during life as a substitute for calcium [74] or if it reflects physiological events, such as maternal transfer since it is a co-factor of the protein FAM20C found in the primordium [76]. Mg and Mn presented similar patterns between years and among areas, with the exception of NSP, which is probably related to environmental conditions and/or physiological events. Rubidium (Rb) usually presents a negative relationship with salinity that was not observed in the *I. parvipinnis* data, possibly due to interactions between Rb, salinity, and physiological processes (e.g., ionic balance or osmoregulatory pathways), interchanging it with K+, that may render Rb more or less available for incorporation into the otolith [71,77,78]. Copper (Cu) is involved in the activity of many essential enzymes and is required in oocyte formation in vertebrates [65]; nevertheless, peaks of Cu and Pb coinciding with the most industrialized locations and under intense port activity (such as CSP and SC in the present study) could be indicative of environmental contamination [79,80].

Unlike other chemical elements, such as Cu, Zn, Ni, Co, and Cr, which are essential in trace amounts but toxic in higher doses, Pb has no known nutritive function in fish, but it is toxic at low levels interfering with essential nutrients of similar characteristics as Ca and Zn [80,81]. However, lead is a trace metal less frequently investigated due to its analytical challenges for quantification [60]. Higher Pb concentrations in 1975 could be a result of the intensified occupation of the coast of the SBB, beginning in the 1950s. Since then, numerous human activities have occurred in the study area, such as sandy extraction from beaches and dunes, modification of river channels, canal construction, dredging, construction of sea walls, and contamination from different sources such as port activity, industries, and domestic sewage discharge [12,82,83,84,85].

The low levels of Pb observed in 2018/2019 compared to 1975 in *I. parvipinnis* otoliths could be the result of the environmental policies that resulted in a worldwide decrease in Pb levels since the early 1980’s due to reduced consumption of leaded petrol [86]. Those temporal variations in Pb concentration were also registered in sediment cores: a reduction in SSP and PR from the 1970s to 2000–2010s [87,88] in contrast to an increase in CSP [89]. The literature also shows the anthropogenic impacts of oil exposure, industrial, gasoline, sewage, and agriculture input not only in Pb concentration in otoliths but also in Ba, Mg, Mn, Na, Sr, and Zn [90,91,92,93,94]. Anthropogenic climate changes, mainly after the 1950s, are also affecting marine biodiversity, ecosystems, fisheries, and ecosystem services [95,96,97]. The literature has already reported evidence of an increase in sea surface temperature in the South Atlantic Ocean mainly after the 1980s [12,98] and its consequences in the ichthyofauna of a transitional zone between the tropical and subtropical regions in Southeastern Brazil [12]. Ocean acidification, warming, and deoxygenation resulting from climate change could influence the ecophysiology of marine organisms and the abundance, distribution, and composition of fish communities [10,99,100]. Once several physiological processes (e.g., ion transport, homeostasis, osmoregulation, growth, or gonad development) are often distressed by those environmental changes, otolith growth and chemistry may also respond to anthropogenic climate changes [59,60,101], explaining the observed long-term changes in the geochemical signatures of *I. parvipinnis*.

Alternative research methods can provide different scenarios about the population structure of marine fishes. A holistic approach is recommended to deal with species and/or geographic areas whose characteristics are not fully understood. An improvement in the reclassification rates has been observed when using otolith elemental signatures compared to otolith shape for several species [9,41,102]. Regarding *I. parvippinis*, those rates slightly increased from whole-body morphometry (79% in 2018/2019; [34]) to otoliths shape (42% in 1975, and 81% in 2018/2019; [33]), and, finally, to otolith chemical composition (58% in 1975, and 80% in 2018/2019; present study). Moreover, compared with the previous data from otolith shape [33], the present study allowed the detection of significant differences between SC and the other locations in both periods, clearly detached it as an isolated population unit for both periods, and increased the regional cross-validation reclassification success.

## 5. Conclusions

The multi-elemental otolith approach showed clear regional differences between *I. parvipinnis* individuals indicating the presence of distinct population units that underwent significant temporal and latitudinal changes. Moreover, the limited connectivity of the SC population with the populations located northwards and the decrease in the degree of adult populations mixing along the SBB over time were also recorded, recommending regional fishery management for the species. The present study also recorded a noticeable anthropogenic interference in fish otoliths fingerprints over a large temporal scale without excluding the potential effects of climate change.

## Figures and Tables

**Table 1:** States, locations, and their respective code, period of capture, sample size (*n*), total length (TL, mm) and otolith mass (OM, mg) of *Isopisthus parvipinnis* used in this study. For TL and OM, mean and standard deviation are presented. Legend: North of São Paulo (NSP), Center of São Paulo (CSP), South of São Paulo (SSP), Paraná (PR), Santa Catarina (SC).

States	Location	Code	1975				2018/2019			
			Period	*n*	TL (mm)	OM (mg)	Period	*n*	TL (mm)	OM (mg)
São Paulo	Ubatuba	NSP	Sep. and Nov.	30	109 ± 12	26.39 ± 6.64	Nov.	30	110 ± 11	30.74 ± 7.38
	Peruíbe	CSP	Sep. and Nov.	30	108 ± 14	27.89 ± 8.14	Nov.	30	134 ± 5	46.29 ± 4.03
	Cananéia	SSP	Sep. and Nov.	30	109 ± 14	28.97 ± 8.67	Oct.	30	113 ± 8	32.95 ± 4.95
Paraná	Paranaguá	PR	Sep. and Nov.	30	109 ± 14	30.06 ± 9.22	Sep.	30	112 ± 11	32.83 ± 7.30
Santa Catarina	Itajaí	SC	Sep. and Nov.	29	111 ± 14	29.45 ± 8.77	May	28	76 ± 10	11.74 ± 4.40

**Table 2:** Two-Way ANOVA comparisons among the five sampling locations regarding the chemical composition of *Isopisthus parvipinnis* otoliths collected in 1975 and 2018/2019. Statistically significant differences (*p* < 0.05) were marked in bold.

	Ba:Ca					Co:Ca				
	DF	SS	MS	F	*p*	DF	SS	MS	F	*p*
Year	1	29,477	29,477	174.20	**<0.000**	1	0.0205	0.0205	0.3514	0.5538
Location	4	17,719	4429.7	26.180	**<0.000**	4	29.862	7.4654	128.20	**<0.000**
Interaction	4	14,975	3743.7	22.130	**<0.000**	4	2.8357	0.7089	12.180	**<0.000**
Within	287	48,560	169.20			287	16.706	0.0582		
Total	296	110,568				296	49.435			
	**Cu:Ca**					**Li:Ca**				
	**DF**	**SS**	**MS**	**F**	***p***	**DF**	**SS**	**MS**	**F**	***p***
Year	1	6.8509	6.8509	45.080	**<0.000**	1	0.2463	0.246	31.740	**<0.000**
Location	4	4.2744	1.0686	7.0320	**<0.000**	4	0.3217	0.080	10.370	**<0.000**
Interaction	4	24.288	6.0720	39.960	**<0.000**	4	0.1510	0.038	4.8650	**<0.000**
Within	271	41.184	0.1520			286	2.2191	0.008		
Total	280	77.142				295	2.9420			
	**Mg:Ca**					**Mn:Ca**				
	**DF**	**SS**	**MS**	**F**	***p***	**DF**	**SS**	**MS**	**F**	***p***
Year	1	9181.7	9181.7	284.800	**<0.000**	1	1851.8	1851.8	101.40	**<0.000**
Location	4	9145.9	2286.5	70.910	**<0.000**	4	2494.5	623.62	34.160	**<0.000**
Interaction	4	5683.1	1420.8	44.060	**<0.000**	4	1708.1	427.03	23.390	**<0.000**
Within	287	9253.7	32.243			287	5239.6	18.256		
Total	296	33,363				296	11,323			
	**Na:Ca**					**Ni:Ca**				
	**DF**	**SS**	**MS**	**F**	***p***	**DF**	**SS**	**MS**	**F**	***p***
Year	1	2.58 × 10^5^	2.58 × 10^5^	1.4630	0.2275	1	7.3276	7.3276	0.6257	0.4296
Location	4	6.40 × 10^6^	1.60 × 10^6^	9.0870	**<0.000**	4	3935.9	983.97	84.020	**<0.000**
Interaction	4	6.60 × 10^6^	1.65 × 10^6^	9.3770	**<0.000**	4	241.86	60.465	5.1630	**<0.000**
Within	287	5.05 × 10^7^	1.76 × 10^5^			287	3361.3	11.712		
Total	296	6.38 × 10^7^				296	7548.7			
	**Pb:Ca**					**Rb:Ca**				
	**DF**	**SS**	**MS**	**F**	***p***	**DF**	**SS**	**MS**	**F**	***p***
Year	1	3.3757	3.3757	788.8	**<0.000**	1	0.0010	0.0010	2.4540	0.1183
Location	4	0.4424	0.1106	25.84	**<0.000**	4	0.0298	0.0074	17.910	**<0.000**
Interaction	4	0.0370	0.0093	2.163	0.0732	4	0.0060	0.0015	3.5890	**<0.000**
Within	287	1.2283	0.0043			287	0.1192	0.0004		
Total	296	5.0685				296	0.1560			
	**Sr:Ca**									
	**DF**	**SS**	**MS**	**F**	***p***					
Year	1	7.18 × 10^6^	7.18 × 10^6^	53.850	**<0.000**					
Location	4	1.97 × 10^7^	4.92 × 10^6^	36.890	**<0.000**					
Interaction	4	3.37 × 10^6^	8.42 × 10^5^	6.3160	**<0.000**					
Within	287	3.83 × 10^7^	1.33 × 10^5^							
Total	296	6.86 × 10^7^								

**Table 3:** Overall and pairwise PERMANOVA comparisons among the five sampling locations regarding the chemical composition of *Isopisthus parvipinnis* otoliths collected in 1975 and 2018/2019. Significant statistical differences (*p* < 0.05) were marked in bold. Legend: North of São Paulo (NSP), Center of São Paulo (CSP), South of São Paulo (SSP), Paraná (PR), Santa Catarina (SC).

Overall PERMANOVA						Pairwise PERMANOVA						
Source	SS	DF	MS	F	*p*		NSP	CSP	SSP	PR	SC	
Year	9.99 × 10^6^	1	9.99 × 10^6^	30.100	**0.0001**	**NSP**		**0.0152**	**0.0001**	**0.0001**	**0.0001**	**1975**
Location	2.74 × 10^7^	4	6.86 ×10^6^	20.676	**0.0001**	**CSP**	**0.0001**		**0.0001**	**0.0002**	**0.0028**	
Interaction	8.23 × 10^6^	4	2.06 × 10^6^	6.1994	**0.0001**	**SSP**	**0.0001**	**0.0002**		0.9031	**0.0001**	
Residual	9.54 × 10^7^	287	3.32 × 10^5^			**PR**	**0.0001**	**0.0001**	0.9367		**0.0001**	
Total	1.40 × 10^8^	296				**SC**	**0.0005**	**0.0001**	**0.0001**	**0.0001**		
							**2018/2019**					

**Table 4:** Jackknifed cross-validation reclassification matrices obtained from otolith’s elemental composition of Isopisthus parvipinnis from all sampling locations for the years 1975 and 2018/2019. Legend: North of São Paulo (NSP), Center of São Paulo (CSP), South of São Paulo (SSP), Paraná (PR), Santa Catarina (SC).

1975						
Original Location	Predicted Location					% Correct
	NSP	CSP	SSP	PR	SC	
**NSP**	21	7	0	2	0	70
**CSP**	12	9	5	4	0	30
**SSP**	0	7	13	10	0	43
**PR**	3	1	11	15	0	50
**SC**	0	0	0	0	29	100
					**Total**	58
**2018/2019**						
**Original** **Location**	**Predicted Location**					**% Correct**
	**NSP**	**CSP**	**SSP**	**PR**	**SC**	
**NSP**	29	0	0	1	0	97
**CSP**	4	26	0	0	0	87
**SSP**	0	2	18	10	0	60
**PR**	1	2	10	17	0	57
**SC**	0	0	0	0	28	100
					**Total**	80

## Data Availability

Not applicable.
